# The role of emotional intelligence and organisational support on work stress of nurses in Ibadan, Nigeria

**DOI:** 10.4102/curationis.v40i1.1715

**Published:** 2017-05-23

**Authors:** Abiodun M. Lawal, Erhabor S. Idemudia

**Affiliations:** 1 Faculty of Social Sciences, Federal University Oye-Ekiti, Nigeria; 2 Faculty of Human and Social Sciences, North-West University, South Africa

## Abstract

**Background:**

Universally, nurses have been reported to be a group at high risk of workplace stress. However, nurses’ responses to stressful situations at work could be the outcomes of individual differences and organisational factors.

**Objectives:**

We examined the independent and joint contributions of four dimensions of emotional intelligence and perceived organisational support in work stress of nurses in a teaching hospital in Nigeria.

**Methods:**

The study was a cross-sectional survey research design, which selected 228 (41 male and 187 female nurses) nurses through the use of convenience sampling. Questionnaires comprising demographics with work stress, organisational support and emotional intelligence scales were administered to the sampled 228 nurses in the study. Data were analysed with the use of correlational matrix and hierarchical multiple regression.

**Results:**

Self-emotion appraisal, others’ emotion appraisal, use of emotion, regulation of emotion and perceived organisational support were found to have joint contributions to explaining work stress among nurses. Others’ emotion appraisal, use of emotion and perceived organisational support were found to have independent relationships with work stress.

**Conclusion:**

Our findings stress that judgement of others’ emotions, accurate use of emotion by nurses and support from management of the hospital are most important in explaining their reactions towards work-related stress.

## Background

The function of nurses in hospitals cannot be overemphasised among other medical personnel. Moreland and Apker (2016) asserted that nurses function as central figures of health teams and that their responsibilities include among others coordination of direct care and communication between team members, patients and their families. In view of this, nurses may be exposed to many demanding responsibilities. It has been consistently documented in the literature globally that nurses experience higher levels of work-related stress (Colff & Rothmann 2014; Kamau, Medisauskaite & Lopes 2015; Okwaraji & Aguwa 2014). According to Colff and Rothmann (2014), nurses experience several job demands that include workloads, health risk posed by contact with patients, fellow workers not doing their jobs, demands from patients and excessive administrative duties.

Furthermore, Sharma et al. (2014) affirmed that work stress brings forth hazardous impacts not only on nurses’ health but also on their ability to cope with job demands, of which adequate attention to patients is of utmost importance. It was reported that 42% of nurses sampled were suffering from moderate to severe levels of stress on the job (Sharma et al. 2014). Okwaraji and Aguwa (2014) reported a high level of emotional exhaustion among 42.9% of a sample of Nigerian nurses and that 53.8% of the respondents experienced reduced accomplishments, 47.6% experienced depersonalisation and 44.1% showed the presence of psychological distress. Similarly, Gandi et al. (2011) reported no gender difference in burnout levels among nurses, but that work–home interference and home–work interference were found as mediators to the relationship between work characteristics and burnout and that the mediational relationship differs between genders. All the aforementioned studies confirm a high presence of work stress in the nursing population.

Apart from other medical personnel and administrative workers, patients are a major category of people that nurses interact with in the hospitals. The interaction between nurses and patients is expected to go beyond only communication of information but also understanding of the patients’ needs and their emotions towards realisation of effective care for the patients. With this in mind, it means it will take an emotionally intelligent nurse to know how to harness together the emotions of patients as well as those of other medical personnel in the hospital. People react to stress in different ways because of several psychological and individual variables. One such variable that is not well documented in the population of Nigerian nurses is emotional intelligence. More importantly, emotional intelligence is for the development of interpersonal and professional competence in nurses (Powell, Mabry & Mixer 2015) and that is why it is seen as helpful in coping with stressful situations. We define emotional intelligence to be the ability of a nurse to perceive accurately, appraise and express emotion in such a way as to be able to manage any perceived challenges in hospitals from fellow medical personnel, administrative officers or the patients.

Many authors have explained emotional intelligence in different dimensions. Some authors have argued five dimensions (Teehan 2006), while others said four dimensions (Law, Wong & Song 2004). In view of this, we explained emotional intelligence in four dimensions in line with Law et al. (2004); and these are self-emotion appraisal, others’ emotion appraisal, use of emotion and the regulation of emotion. Self-emotion appraisal is the ability to understand and express one’s own emotion. Others’ emotion appraisal is the ability to perceive and understand the emotions of others. Use of emotion is the ability to use one’s emotions effectively by directing them towards constructive activities and personal performance. Regulation of emotion is the ability to manage one’s own emotions (Law et al. 2004).

Many studies have documented that emotional intelligence relates to or affects experience of work stress among nurses (Kalyoncu et al. 2012; Karimi et al. 2014; Nel, Jobker & Rabie 2013; Powell et al. 2015). Specifically, Nel et al. (2013) reported that a relationship exists between emotional intelligence and job characteristics, work wellness, within a nursing environment. Both emotional labour and emotional intelligence were reported to have effects on well-being and job stress among nurses, with emotional intelligence playing a moderating role in the experience of job stress (Karimi et al. 2014). Powell et al. (2015) recently reported that correlations exist between emotional intelligence and better overall health, increased work satisfaction, higher well-being and decreased risk of job burnout in nursing. In view of this, the importance of emotional intelligence in reducing work stress cannot be overemphasised in the nursing population. This suggests that nurses who are high in emotional intelligence may cope better with stressful situations at work while those who are low in emotional intelligence may cope poorly with stressful situations.

Another factor that may be germane in the experience of work-related stress by nurses is availability of support from management of hospitals. The importance of organisational support in work stress was substantiated in many studies (Higazee, Raya & Khalil 2016; Nie & Zhang 2013; Thorsteinsson, Brown & Richards 2014). To affirm the importance of a supportive environment in reducing health-related consequences, of heightened presence of stress among workers, Thorsteinsson et al. (2014) investigated the relationships between work stress, perceived organisational support, supervisor support, staff health and work outcomes and found that high work stress was associated with health-related indicators such as depression, anxiety and fatigue as well as work outcomes such as greater turnover intention. In the same study, less workplace support was found to be associated with adverse work outcomes and a high level of depression (Thorsteinsson et al. 2014). Santos et al. (2003) reported that apart from nursing itself, organisational and management characteristics influence the stress nurses experience at work. In another study, Eze (2014) found that nurses with low organisational support experienced a higher level of burnout than those with high organisational support. In line with these previous findings is the assertion that a large part of potential sources of stress for nurses appear to be organisational in nature; this could be physical, psychological or social in nature (Duquette et al. 1994; Santo et. al. 2003). The study fills the gaps in the scarcity of work stress literature in Nigerian nurses and helps in understanding how dimensions of emotional intelligence in nurses and organisational support might help in explaining work stress. The aim of the study was to examine independent and joint contributions of four dimensions of emotional intelligence and perceived organisational support on work stress of selected nurses in a Nigerian teaching hospital.

## Theoretical models guiding the study

### Transactional model of stress

He transactional model hads long been identified as a cogent theory widely used to explain stress (Antonovsky 1979; Lazarus 1966). The model is based on the underlying process of interaction between an individual and the environment. A fundamental idea about the transactional model of stress is the individual’s cognitive assessment of the demands made on workers and their perceived abilities to cope with those demands (Lazarus 1966; Lazarus & Folkman 1984). In other words, an individual may experience stress when the demands made on him or her surpass his or her capability. In this context, nurses experience stress on the job when they perceive the demands of the job to outweigh their abilities. Nevertheless, what one person finds stressful can change over time and in different instances. However, cognitive assessment of job demand and an individual’s ability to deal with it may depend on certain moderating factors such as the person’s personality or disposition, previous experience, age, sex and coping skills, just to mention a few. The model also emphasises that a person can experience stress physiologically, psychologically, behaviourally as well as socially, which in the long run could be detrimental to both the individual concerned and the organisation where he or she works.

### Job demand – Control support theory

Another relevant theoretical model we used to guide the study is the Job Demand-Control (JDC) Support Model (Karasek & Theorell 1990), which is an extension of the original JDC model (Karasek 1979). The JDC has long been used to explain occupational stress-related research. The model is based on the premise that job strains result from the interaction between two dimensions of the work environment, that is, *psychological job demands* and *job control.* Conventionally, psychological demands are seen to include workloads, time pressure and role conflict. Cognitive and emotional demands as well as interpersonal conflict have been included lately in the explanation of what we call psychological demands (Karasek et al. 1998). Job control is seen as a person’s ability to control his or her work activities in terms of making decisions on the job and using skill discretion on the job (Theorell & Karasek 1996). Practically, using nurses as an example, this model proposes that those who experience high demands on their job coupled with low control over work activities are more likely to report work-related stress, which may result in poor physical and psychological health in the long run. However, social support was later included as a social dimension of the JDC model (Johnson & Hall 1998; Karasek & Theorell 1990) and this was reported to have a moderating effect on the negative impact of job-related tension or pressure on workers’ physical and mental health. For the purpose of this study, availability of support from management of the teaching hospital would serve as a moderator to any unanticipated work-related stress that nurses might be experiencing at work.

## Method

### Research design

This was a cross-sectional study that utilised survey research design. The independent variables were the four dimensions of emotional intelligence (self-emotion appraisal, others’ emotion appraisal, use of emotion and the regulation of emotion) and perceived organisational support, while the dependent variable was work stress.

### Population and setting

The population of the study comprised nurses in University College Hospital (UCH), located in Ibadan, Oyo state, in the south-western part of Nigeria. UCH is a federal government-owned teaching hospital attached to the University of Ibadan (Ibadan 2014). It was established by an Act of Parliament in November 1952 in response to the need for the training of medical personnel and other healthcare professionals for the country and the west African subregion.

The hospital commenced its operations in 1953. The care system of the hospital is public and focuses on general health, teaching and research. Currently, the UCH has about 850 bed spaces for clients. The population of nurses in the clinical nursing department is about 1303 cutting across all cadres of the profession

### Sample and sampling method

Participants were nurses of rank Nursing Officer II, Nursing Officer I, Senior Nursing Officer, Assistant Chief Nursing Officer and Chief Nursing Officer in UCH. A convenience sampling method was used in which nurses who volunteered to participate in the study were given questionnaires to complete. This is a non-probability sampling technique that involves selection of participants who are close to hand and give consent to participate in a study. To arrive at the sample size, we used Slovin’s formula: *n* = *N*/(1 + *Ne*^2^), where *n* = sample size, *N* = population and *e* = error tolerance. With a population of 1303 nurses and 0.06 as error tolerance, we were able to arrive at the sample size of 228 for the study. In practice, 1303/(1 + (1303 * (0.06 * 0.06)) = 1303/(1+ (1303*0.0036) = 1303/(1 + 4.69) = 1303/5.69 = 228.99 = 228. With this result, 228 nurses completed the questionnaires and they comprised 41 male and 187 female nurses. The participants’ ages ranged from 21 to 60 years (*M* = 32.59 years; SD = 8.10). Distribution of respondents’ demographic profile is presented in Table 1.

**TABLE 1:** Respondents’ demographic profile.

Variable	*N* = 228	%
Sex		
Male	41	18.00
Female	187	82.00
Marital status		
Single	89	39.00
Married	127	55.70
Separated	5	2.20
Widow	7	3.10
Religious affiliation		
Christianity	174	76.30
Islam	52	22.80
Others	2	0.90
Educational qualification		
Postgraduate	12	5.30
RN + Post basic/HND	114	50.00
RN	55	24.10
First degree	47	20.60
Rank/job position		
NO II	95	41.70
NO I	64	28.14
SNO	29	12.70
ACNO	22	9.60
CNO	18	7.90

RN, registered nurse; NO II, nursing officer II; NO I, nursing officer I; SNO, senior nursing officer; ACNO, assistant chief nursing officer; CNO, chief nursing officer; HND, higher national diploma.

### Instrument

*Work stress* was assessed using the Workplace Stress Scale (WSS) developed by the Marlin Company, North Haven, CT, USA, and the American Institute of Stress, Yonkers, NY, USA (2001). The WSS consists of eight items describing how often a respondent feels an aspect of his or her job. Examples of items in the scale include ‘Conditions at work are unpleasant or sometimes even unsafe’ and ‘I feel that my job is negatively affecting my physical or emotional well-being’. In terms of scoring, item numbers 6, 7 and 8 are reverse-scored. The scale is in the five-point Likert response format, ranging from never (scored 1) to very often (scored 5). High scores are indicative of higher levels of job stress. Respondents’ total scores are interpreted as follows: Scores of 15 and below are interpreted as relatively calm, 16–20 is interpreted as fairly low in work stress, 21–25 is interpreted as experiencing moderate levels of work stress, 26–30 is interpreted as experiencing severe levels of work stress and 31–40 is interpreted as experiencing a potentially dangerous level of work stress. We assessed the validity of the scale by seeking opinions of nurses as experts. Their suggestions were discussed by the authors and considered in the modification of test items where necessary. In the current study, we reported a Cronbach’s alpha reliability coefficient of 0.70 for the entire scale WSS.

*Organisational support* was assessed using the Perceived Organisational Support scale (POS) developed by Eisenberger et al. (1986). The scale consists of eight items measured by a six-point Likert response ranging from strongly disagree (scored 1) to strongly agree (scored 6). Examples of items in the POS scale include ‘My organisation really cares about my well-being’ and ‘My organisation would forgive an honest mistake on my part’. High scores are indicative of higher perceptions of organisational support. As on the perceived WSS, we assessed validity of this scale by seeking opinions of nurses as experts. Their suggestions were discussed by authors and considered in the modification of test items where necessary. In the current study, we reported a Cronbach’s alpha reliability coefficient of 0.89 for the scale.

*Emotional intelligence* was assessed by the 16-item Wong and Law Emotional Intelligence Scale (WLES) by Wong and Law (2002). Examples of items in the scale include ‘I have a good sense of why I have certain feelings most of the time’ and ‘I have a good understanding of my own emotions’. The WLES was based on the four-dimensional definition of emotional intelligence of Davies, Stankov and Roberts (1998); therefore, the WLES has four subscales with each subscale measured with four items as follows: *self-emotion appraisal (*items, 1, 2, 3 and 4); *others’ emotion appraisal* (items 5, 6, 7 and 8); *use of emotion* (items 9, 10, 11 and 12) and *regulation of emotion* (items 13, 14, 15 and 16). The scale is rated on a six-point Likert response ranging from strongly disagree (scored 1) to strongly agree (scored 6). High scores imply higher emotional intelligence. In the current study, we reported a Cronbach’s alpha reliability coefficient of 0.89 for the scale.

### Data collection procedure

The respondents were informed about the study, aim and research objectives prior to agreeing to complete the questionnaires. In order to ensure confidentiality of responses given to items inside the questionnaire, the respondents were told not to include their names or any form of identification on the self-administered questionnaires. With the help of research assistants, we distributed the questionnaires to respondents. The questionnaires were collected from each respondent immediately after completion and each respondent was thanked for completing the questionnaires. A total of 300 questionnaires were distributed; 252 were retrieved, indicating a response rate of 84%. However, in order to meet the sample size, 228 questionnaires that were properly completed were used for data analyses in the study. This suggests that 24 questionnaires that were not properly completed were discarded.

### Data analyses

Data were analysed using IBM SPSS Statistics version 24. In our analyses, we conducted both descriptive and inferential statistics to present results for the study. Demographic characteristics were analysed with the use of descriptive statistics such as mean, standard deviation and frequencies. We first conducted intercorrelation analysis to determine relationships among variables of interest in the study. Secondly, we used hierarchical multiple regression analyses to determine independent and joint contribution of predictor variables to explain levels of work stress in nurses. Statistics were significant at 0.01 and 0.05 levels of significance.

### Ethical consideration

Institutional ethical clearance and permission were obtained from the Research and Ethics Committee, Department of Psychology, Federal University Oye-Ekiti, Nigeria. Permission to conduct the research was further sought from the head of the clinical nursing department. All participants were informed of the study objectives and they indicated their consent by ticking the ‘Yes’ portion of the front page of the questionnaires. Confidentiality of information divulged in the questionnaires was assured, and participants were promised that the information would be used only for research purposes. Participants were informed that they could opt out of the study if they wished to do so at any time. No monetary incentives were offered to participants. Therefore, administration of questionnaires was made voluntary without any form of imposition. After the completion of questionnaires, individual nurses were thanked for participating in the study.

## Results

Considering the scoring and interpretation reported for the use of WSS, where respondents with scores ranging from 21 to 25 are regarded as experiencing moderate levels of workplace stress, we reported that the group of nurses (*M* = 22.27) sampled in the current study were undergoing moderate levels of work stress. More importantly, it was found that some respondents in the sample reported a score of up to 37.00, which indicates a work stress level that is potentially dangerous to an individual’s health. Generally, the nurses’ scores suggest that certain characteristics about their job appear to be stressful. General emotional intelligence and perceived organisational support levels in the group of respondents sampled in this study (*M* = 74.41) indicate that nurses possess higher general emotional intelligence. Also, the mean score on organisational support (*M* = 25.47) indicates higher perceived organisational support. Observation of the maximum score reported by some respondents in each of these variables asserts the presence of emotional intelligence (Max. *=* 95.00) and organisational support (Max. *=* 44.00) in the sample.

Before conducting a hierarchical multiple regression analysis, we tested the relevant assumptions of this statistical analysis by taking some scientific processes. Firstly, the study meets the assumption on sample size because a minimum sample size of 96 was reported adequate for including five predictor variables in the analyses (Tabachnick & Fidell 2001). Our study is deemed adequate with a sample size of 228. Our study also meets the assumption of singularity of independent variables, as self-emotion appraisal, others’ emotion appraisal, use of emotion, regulation of emotion and perceived organisational support were not a combination of other independent variables or one another. Also, scrutinising the intercorrelational matrix analysis (see Table 2), one could see that none of the predictor variables was highly correlated with the criterion variable; rather all were within accepted limits with the exception of self-emotion appraisal, which was not correlated significantly with work stress.

**TABLE 2:** Mean, standard deviation and correlational matrix between variables in the study (*N* = 228).

No.	Variable	1	2	3	4	5	6
1	Self-emotion appraisal	-	-	-	-	-	-
2	Others’ emotion appraisal	0.26*	-	-	-	-	-
3	Use of emotion	0.49*	0.44*	-	-	-	-
4	Regulation of emotion	0.35*	0.48*	0.62*	-	-	-
5	Organisational support	−0.09	0.15	−0.06	0.05	-	-
6	Work stress	−0.08	−0.23*	−0.20*	−0.24*	0.31*	-
	*M*	19.53	18.10	20.77	19.82	25.47	22.27
	*SD*	3.29	3.93	3.25	3.30	8.51	4.95

M, mean; SD, standard deviation.

* , *p* < 0.01.

We first conducted intercorrelation analysis of relevant variables in the study, which comprised the four dimensions of emotional intelligence: *self-emotion appraisal, others’ emotion appraisal, use of emotion* and *regulation of emotion*, perceived organisational support and work stress. Results of the correlational matrix in Table 2 show that self-emotion appraisal does not correlate (*r* = -0.08; *p* > 0.01) with work stress, but others’ emotion appraisal (*r* = -0.23; *p* < 0.05), use of emotion (*r* = -0.20; *p* < 0.01) and regulation of emotion (*r* = -0.24; *p* < 0.01) negatively correlated significantly with work stress of nurses. The interpretations of these results imply that self-emotion appraisal in nurses does not relate to their level of work stress. However, nurses who can accurately appraise other people’s emotions, who use emotion appropriately and who can regulate emotion experience less work stress. Perceived organisational support (*r* = -0.20; *p* < 0.01) negatively correlated significantly with work stress of nurses. This can be interpreted to mean that the more nurses perceive support from their management or organisation, the less they report work stress.

We further conducted a hierarchical multiple regression of five models in order to explore how well each of the predictor variables predicted level of work stress of nurses (see Table 3). Perceived organisational support was first entered at stage 1 of the regression, self-emotion appraisal was entered at stage 2, others’ emotion appraisal at stage 3, use of emotion at stage 4 and regulation of emotion at stage 5. Predictor variables were entered in this order as the dimensions are presented in the emotion intelligence scale and more importantly based on the sequential likelihood that there is a need for availability support from place of work, then an individual needs to understand his or her emotion, followed by understanding other people’s emotions, then know how to use the emotion effectively and be able to regulate the emotion.

**TABLE 3:** Summary of hierarchical regression analysis for variables predicting work stress of nurses (*N* = 228).

Variable	Model 1			Model 2			Model 3			Model 4			Model 5		
				
	B	SEB	*β*	B	SEB	*β*	B	SEB	*β*	B	SEB	*β*	B	SEB	*β*
Organisational support	−0.18	0.04	-	−0.31	−0.19	−0.32	−0.17	0.04	−0.29**	−0.18	0.04	−0.31**	−0.17	0.04	−0.30**
Self-emotion	-	-	-	-	−0.16	−0.11	−0.09	0.10	−0.06	0.01	0.11	0.01	0.02	0.11	0.01
Others’ emotion	-	-	-	-	-	-	−0.21	0.08	−0.17*	−0.13	0.09	−0.11	−0.09	0.09	−0.07
Use of emotion	-	-	-	-	-	-	-	-	-	−0.27	0.12	−0.18*	−0.17	0.13	−0.11
Regulation of emotion	-	-	-	-	-	-	-	-	-	-	-	-	−0.19	0.12	−0.13
*R*	-	0.31	-	-	0.22	-	-	0.36	-	-	0.39	-	-	0.40	-
*R*^2^	-	0.09	-	-	0.11	-	-	0.13	-	-	0.15	-	-	0.16	-
Adjusted *R*^2^	-	0.09	-	-	0.10	-	-	0.12	-	-	0.14	-	-	0.14	-
*R*^2^ Change (%)	-	0.09	-	-	0.01	-	-	0.03	-	-	0.02	-	-	0.01	-
*F*	-	24.07**	-	-	13.59**	-	-	11.42**	-	-	10.04**	-	-	8.58**	-
*F* Change	-	24.07**	-	-	2.91	-	-	6.42*	-	-	5.25*	-	-	2.48	-

B, unstandardised coefficient; SEB, standard error of the computed value of B; *β*, standardised coefficient; *R*, correlation between observed value and the predicted value of work stress; *R*^2^, square of R; *F*, ratio of the mean regression.

* , *p* < 0.5;

** , *p* < 0.01.

The hierarchical multiple regression revealed that at stage 1, perceived organisational support contributed significantly to the regression model, *F* (1226) = 24.07, *p* < 0.01, and accounted for 9.6% of the variation in work stress. Introducing self-emotion appraisal as one of the four dimensions of emotional intelligence, we found that the variable explained an additional 1.2% of variation in work stress although the change in *R*^2^ was not significant, *F* (2225) = 13.59, *p* < 0.01. At stage 3, adding others’ emotion appraisal to the regression model contributed significantly and explained an additional 2.5% of the variation in work stress. This change in *R*^2^ was significant, *F* (3224) = 11.42, *p* < 0.01. At stage 4, we added use of emotion to the regression model and it contributed significantly and also explained an additional 2% of the variation in work stress. This change in *R*^2^ was significant, *F* (4223) = 10.04, *p* < 0.01. Finally, the addition of regulation of emotion to the regression model explained an additional 0.9% of the variation in work stress and this change in *R*^2^ was not significant, *F* (2222) = 8.58, *p* < 0.01. When all five predictor variables were included in stage 5 of the regression model, only perceived organisational support was a significant predictor of work stress, while all the four dimensions of emotional intelligence were not significant predictors of work stress of nurses. Together, all the five predictor variables accounted for 16.2% in the variation of work stress, out of which perceived organisational support accounted for 9.6% and the four dimensions of emotional intelligence jointly accounted for 6.6%.

## Discussion

In this study, the level of work stress among nurses working in a teaching hospital in Nigeria was determined. Work stress continues to be an issue of concern in the nursing profession. Hence, we examined four dimensions of emotional intelligence and perceived organisational support as possible predictors of work stress among nurses. Our findings revealed a moderate level of work stress being experienced by the nurses sampled, though with indication that few of these nurses might be experiencing some form of stress level that could be potentially dangerous. Generally, our finding corroborates other previous studies carried out within and outside Nigeria, which have documented the presence of some degree of work stress among nurses (Colff & Rothmann 2014; Kamau et al. 2015; Okwaraji & Aguwa 2014). The presence of some form of stress being experienced by nurses could be attributed to the nature of the profession itself. Many times, nurses are confronted with some tasks that are routine and even frightening. Other tasks assigned to nurses that might pose a threat to their well-being include excessive noise in the wards or undue quiet, unpleasant sights and sounds, time pressure, standing for long periods and so forth. All these experiences could be enough to subject nurses to a heightened level of stress at their place of work.

In the same study, we found that perceived organisational support and the four dimensions of emotional intelligence *self-emotion appraisal, others’ emotion appraisal, use of emotion* and *regulation of emotion* jointly contributed significantly in explaining the level of work stress of nurses. Apart from looking at the joint contribution of all five independent variables, we statistically used hierarchical regression to examine independent contribution of each of the predictor variables in the explanation of work stress of nurses. In the first model, perceived organisational support was found to be significantly negatively associated with work stress. Specifically, nurses who perceived more support from the management reported less work stress compared to those who perceived less support. The finding that nurses with more organisational support report less work stress relative to those with less organisational support shows the importance of the availability of support from the management of hospitals in stress reduction processes for nurses. Our finding is consistent with previous work carried out that has affirmed the importance of organisational characteristics (Duquette et al. 1994). Specifically, our finding is in line with the finding that organisational support is relevant in the management of and coping with stressful situations among nurses or in the nursing profession (Santo et al. 2003; Thorsteinsson et al. 2014) and that organisational support influences the level of burnout among nurses (Eze 2014). We believe that organisational support cannot be ignored in the stress management process for the nurses because when nurses perceive such support from their management, they feel their contributions to the hospitals are valued and appreciated and that their welfare is well taken care of. More importantly, nurses would refer to availability of support they receive from management of hospital as vital in their well-being. Another standpoint to establish the importance of organisational support in reducing work stress could be linked to the idea of social exchange theory that argues that nurses as individuals would be willing to exchange their efforts and time in taking care of patients for various rewards or supports offered to them by the organisation.

In the second model, we added self-emotion appraisal. Although our finding revealed joint contribution of organisational support and self-emotion appraisal in explanation of work stress, self-emotion appraisal did not have independent relationship with work stress of nurses. It suggests that self-awareness of emotion by nurses is not effective by itself in coping with stressful situations at work, but is in conjunction with other facets of emotional intelligence coupled with availability of support from management of the hospital. Others’ emotion appraisal was added to the regression model and a joint contribution of organisational support, self-emotion appraisal and others’ emotion appraisal was found. Also, organisational support and others’ emotion appraisal have independent contributions to the explanation of the level of work stress of nurses. Interestingly, apart from the joint contribution, others’ emotion appraisal was found to have a negative relationship with work stress. This is an indication that for nurses to experience less work-related stress, they need to understand the emotions of other significant people around them, which includes patients.

In the fourth model, use of emotion was added and this also led to the joint contribution of the predictor variables and an independent negative relationship between use of emotion and work stress. The finding indicates for nurses to enjoy less work stress, they need to understand how to use and channel their emotions accurately and constructively. Finally, regulation of emotion was added to the previous predictor variables and joint contribution was recorded. However, an independent relationship between regulation of emotion and work stress was not observed. It suggests that knowing how to manage emotions may not be effective by itself in dealing with work-related stress, unless this is harnessed along with other dimensions of emotional intelligence with support from the management. Our findings are in line with many previous studies that have confirmed the importance of emotional intelligence in work stress among nurses (Karimi et al. 2014; Nel et al. 2013; Powell et al. 2015). Perhaps the relevance of emotional intelligence in reducing work stress could be traced to the fact that being high in emotional intelligence may enable nurses to, for instance, perceive emotions, assimilate any form of feelings in patients and fellow medical personnel, understand the information of those emotions and be able to manage them well.

### Implications of major findings and recommendations

The finding that all four dimensions of emotional intelligence relate with work stress among nurses implies that nurses who have the skills to understand their own emotions as well as others’ can easily link these emotions together in managing any form of work-related stress, whether from their fellow medical personnel or from their patients. Also, the finding that perceived organisational support relates with work stress implies that nurses tend to cope better with work-related stress when they receive some form of support from the management of the hospital. In general, we recommend that there should be continual individual and organisation-focused stress management interventions. Presence of individual-focused stress management intervention for nurses will enhance their abilities to cope with likely work-related difficulties, especially when the nurses are educated on what stress is, its nature, sources, effects and health-related implications. This type of intervention can help reduce the likely indicators of stress among nurses. Because it was found in this study that emotional intelligence is important, we recommend that the management of hospitals should continually organise trainings and workshops for nurses on how to enhance their abilities to recognise and regulate emotions in them and in others in order to manage any stressful situations they encounter in their workplace. Our recommendation on the importance of organisational support in reducing work stress among nurses is that management of the teaching hospital should make efforts to provide all necessary facilities that can ensure adequate care for nurses’ welfare when providing general overwhelming healthcare services.

### Limitations of the study

Undoubtedly, the study was confronted with lots of limitations that need to be addressed in further studies that have a similar focus. One of the major limitations of the study was the use of a questionnaire as a self-report assessment tool designed to tap information on variables in the study. We report this as a limitation because respondents can fake their responses in order to present themselves in a desired manner, which could affect the general opinion of the subject matter in the population sampled. To avoid this in the future, additional methods of data collection such as observation of nurses’ workloads and supervisor’s rating of individual nurses’ workloads and performance could help in having an in-depth knowledge of variables of concern in this study. Also, the study was confronted with the limitation of using a small sample, which could question the true representation of the number of nurses sampled in the teaching hospital or using more than one hospital as the setting. A larger sample size of nurses could make the findings more generalisable. Nevertheless, our study has been able to confirm the presence of work stress in a nursing population as well as the importance and relevance of emotional intelligence and organisational support in reducing work stress or as vital ingredients in stress management procedures for the nursing population.

## Conclusion

In view of the finding that perceived organisational support consistently contributed significantly to the explanation of work stress among nurses, we concluded that when various forms of support are adequately provided to nurses by the management of hospitals, the tendency to report higher levels of work stress by the nurses will be reduced. Also, we found that all four dimensions of emotional intelligence along with perceived organisational support contributed jointly in explaining work stress of nurses. However, understanding others’ emotions and constructive use of emotion by nurses appear to be more relevant in managing work stress. More importantly, the capacity to understand and reason about others’ emotions and be able to positively use emotion is important for nurses in teaching hospitals to enhance their thoughts, knowledge and intellectual growth to be able to attend to patients and colleagues cordially.
